# Mechanisms of Cisplatin-Induced Apoptosis and of Cisplatin Sensitivity: Potential of BIN1 to Act as a Potent Predictor of Cisplatin Sensitivity in Gastric Cancer Treatment

**DOI:** 10.1155/2012/862879

**Published:** 2012-06-12

**Authors:** Satoshi Tanida, Tsutomu Mizoshita, Keiji Ozeki, Hironobu Tsukamoto, Takeshi Kamiya, Hiromi Kataoka, Daitoku Sakamuro, Takashi Joh

**Affiliations:** ^1^Department of Gastroenterology and Metabolism, Graduate School of Medical Sciences, Nagoya City University, 1 Kawasumi, Mizuho, Nagoya, Aichi 467-8601, Japan; ^2^Department of Pathology, Stanley S. Scott Cancer Center, Health Science Center, Louisiana State University, CSRB, 533 Bolivar Street, New Orleans, LA 70112, USA

## Abstract

Cisplatin is the most important and efficacious chemotherapeutic agent for the treatment of advanced gastric cancer. Cisplatin forms inter- and intrastrand crosslinked DNA adducts and its cytotoxicity is mediated by propagation of DNA damage recognition signals to downstream pathways involving ATR, p53, p73, and mitogen-activated protein kinases, ultimately resulting in apoptosis. Cisplatin resistance arises through a multifactorial mechanism involving reduced drug uptake, increased drug inactivation, increased DNA damage repair, and inhibition of transmission of DNA damage recognition signals to the apoptotic pathway. In addition, a new mechanism has recently been revealed, in which the oncoprotein c-Myc suppresses bridging integrator 1 (BIN1), thereby releasing poly(ADP-ribose)polymerase 1, which results in increased DNA repair activity and allows cancer cells to acquire cisplatin resistance. The present paper focuses on the molecular mechanisms of cisplatin-induced apoptosis and of cisplatin resistance, in particular on the involvement of BIN1 in the maintenance of cisplatin sensitivity.

## 1. Introduction

Gastric cancer is one of the most prevalent malignancies in Japan and East Asian countries [1], This cancer represents the major cause of mortality in these countries, despite great advances in diagnosis and multimodal treatments [2]. Cisplatin (cis-Diamminedichloroplatinum (II): CDDP) is an important chemotherapeutic agent in the treatment of advanced gastric cancer. Some trials of combination chemotherapy with S-1 and cisplatin as the first-line or second-line treatment for advanced and recurrent gastric cancer have yielded good responses and this treatment is well tolerated [3, 4]. However, there is diversity in the efficacy of cisplatin and in patient response to anti-cancer drugs including cisplatin, which can be of importance in terms of therapeutic outcome. Molecules and factors that are capable of predicting patient responses and resistance to anticancer agents are therefore of great interest and have been extensively studied. A recent study of the potent predictor of cisplatin sensitization Bridging integrator 1 (BIN1, also known as box-dependent MYC-interacting protein 1) demonstrated that BIN1 plays an important role in sensitization to cisplatin [5]. BIN1 is a nucleocytoplasmic adaptor protein that is involved in pleiotropic cellular functions such as suppression of oncogenic transformation. Here, we provide an overview of the molecular mechanism of cisplatin-induced apoptosis and of cisplatin resistance as well as of the mechanisms by which BIN1 sensitizes cancer cells to cisplatin.

## 2. Cisplatin and Its Stereoisomer Transplatin

Cisplatin was first discovered in 1965 as a strong inhibitor of bacterial cell growth and some years later was found to be a potent antitumor drug in studies using the murine leukemia L1210 cell line [8, 9]. Cisplatin is actually one of the most widely used anticancer drugs, and the central role of this drug in human cancer chemotherapy attests to its current importance. Cisplatin-based chemotherapy is highly efficient for the treatment of patients with a variety of cancers such as lung, ovarian, head and neck, and gastric cancer [3, 10]. On the other hand, its trans analogue, transplatin, is known to be biologically inactive because of the diversity of qualitative and quantitative DNA adducts that it forms compared with cisplatin [6].

## 3. Molecular Mechanisms of Cisplatin-Induced Pro-Apoptotic Effects

### 3.1. DNA Strand-Crosslinks and DNA Damage Recognition

Cisplatin exerts its cytotoxic properties by reacting with DNA, which eventually culminates in irreversible apoptosis. Cisplatin primarily interacts with the N7-sites of purine residues in DNA to form DNA-DNA interstrand and intrastrand crosslinks [11]. The intrastrand adducts, ApG and GpG in particular, are responsible for the cytotoxic effects of cisplatin and account for 85–90% of the bound platinum [12]. These adducts block DNA replication and transcription. DNA adduct formation is followed by DNA damage recognition by over 20 proteins including hMSH2 of the mismatch repair (MMR) complex, the nonhistone chromosomal high-mobility groups 1 and 2 (HMG1 and 2) proteins and the transcriptional factor “TATA-binding protein” (TBP) [13–15]. The putative role of these DNA damage recognition proteins is to transmit DNA damage signals to downstream signaling cascades involving p53, MAPK, and p73, which ultimately induce apoptosis.

### 3.2. Cisplatin-Induced p53 and MAPK Activation

As mentioned above, cisplatin is believed to mediate activation of the p53 protein, a tumor suppressor, following DNA damage recognition. The transcriptional activation and stability of the p53 protein is known to be regulated by the two kinases ataxia telangiectasia-mutated protein (ATM) and ATM- and Rad3-related protein (ATR) [16, 17]. Cisplatin preferentially activates the ATR kinase which phosphorylates p53 on serine-15, resulting in its activation [18]. Of the mitogen-activated protein kinase (MAPK) signals that are involved in cisplatin-induced toxic effects, including extracellular signal-related kinases (ERKs), c-Jun N-terminal kinases (JNKs), and the p38 kinases, ERK activation appears to be the most important since activated ERK also phosphorylates p53 on serine-15 [19]. In addition, activation of JNK/p38 results in phosphorylation of the transcription factors c-Jun and activating transcription factor (ATF)-2 which, in turn, can bind to AP-1 binding sites in the promoters of multiple target genes. This cascade ultimately induces apoptosis through proapoptotic FasL gene expression.

### 3.3. Cisplatin Activation of p73-Dependent Apoptotic Signaling

**Figure 1:**
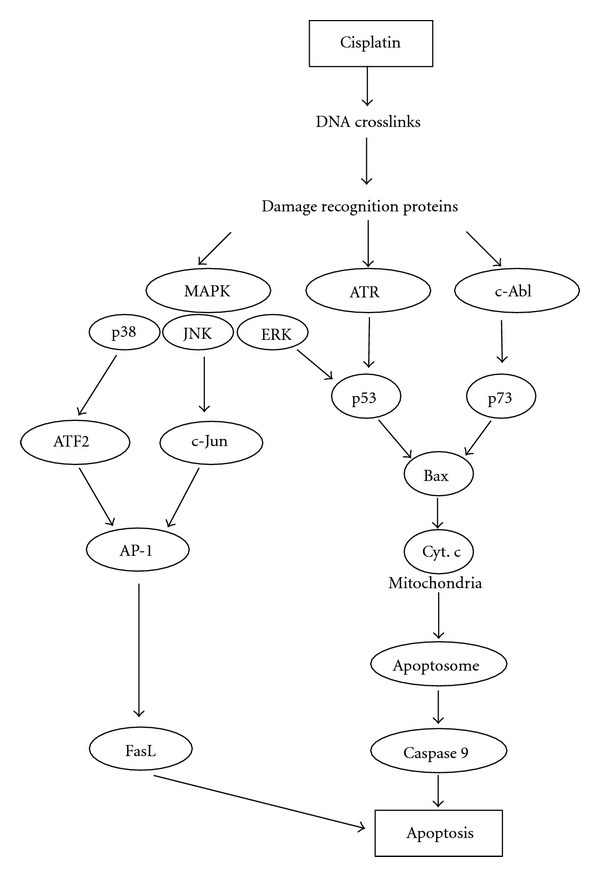
Signal transduction cascades mediating cisplatin-induced apoptosis. Quoted from [6] and modified.

 p73 is a nuclear p53-related protein, which functions as a pro-apoptotic protein and accumulates in cisplatin-treated cells. Cisplatin-induced accumulation of p73 is dependent on MLH1 MMR proteins since it does not occur if the cells are deficient in these proteins [20]. Cisplatin-induced activation of the MMR protein-dependent p73 cell death pathway differs from its activation of p53 in that p73 activation does not involve ATR phosphorylation. The link between DNA damage recognition protein and activation of p73 may be the oncogenic tyrosine kinase c-Abl. Thus, cisplatin activates c-Abl, and accumulation of p73 following cisplatin treatment is not observed in c-Abl-defective cells. Cisplatin activation of c-Abl is regulated by MMR because MLH1-defective cells fail to activate c-Abl during cisplatin treatment. Furthermore, cisplatin cytotoxicity is reduced in c-Abl defective cells [21, 22]. The likely downstream events of both the cisplatin-induced p73 and p53 pathways are that cytochrome c is released through the mitochondrial membrane via Bax and Bak induction [23], which ultimately results in apoptosis through caspase 9 activation [6, 24] (Figure 1).

### 3.4. Cisplatin Modulation of Cell Cycle Checkpoints

 Cisplatin-induced DNA damage induces an initial transient S-phase arrest, which is followed by inhibition of Cdc2-cyclin A or B kinases to yield a persistent G2/M arrest [25, 26]. As the inhibitory effect of cisplatin on the G1-phase cyclin-dependent kinases is a later event in the cell cycle checkpoint, accumulation of cells in the G1 phase is seldom observed and the cells remain in the G2/M phase.

## 4. Mechanisms of Cisplatin Resistance

To date, the mechanisms of cisplatin resistance have been suggested to involve reduced intracellular cisplatin accumulation, increased inactivation of cisplatin by thiol-containing molecules, increased DNA damage repair, and inhibition of transmitted DNA damage recognition to apoptotic pathways.

### 4.1. Reduced Intracellular Drug Accumulation

Reduced cisplatin accumulation in cells is caused by either inhibited drug uptake or increased drug efflux. Regarding inhibited drug uptake, active transporters such as Na^+^K^+^-ATPase or a gated ion channel are involved in cisplatin uptake. The inactivation and down-regulation of these uptake transporters result in cisplatin-resistance [27, 28]. Regarding increased cisplatin efflux, MRP2, which is one of the 7 known isoforms of the multidrug resistance-associated protein (MRP) family, appears to be important for cisplatin resistance. An increased level of this transporter protein was observed in resistant cells [29]. Furthermore, antisense depletion of MRP2 increased cisplatin sensitivity, supporting the involvement of MRP2 in cisplatin resistance [30]. Another transporter that is involved in cisplatin efflux is the protein encoded by the copper-transporting P-type ATPase gene, ATP7B, which mediates resistance to both copper and cisplatin. High levels of ATP7B mRNA in ovarian cancer correlated with cisplatin resistance. It has been also proposed that ATP7B expression is useful as a clinical marker of cisplatin resistance [31].

### 4.2. Increased Cisplatin Inactivation by Thiol-Containing Molecules

Glutathione, (gamma-glutamylcysteinylglycine: GSH) which is the most abundant intracellular thiol, can detoxify many toxins including cisplatin. Cisplatin can be catalytically converted into cisplatin-thiol conjugates by GSH-S-transferase *π*, and these conjugates are ultimately inactivated [32].

### 4.3. Increased DNA Damage Repair

**Figure 2:**
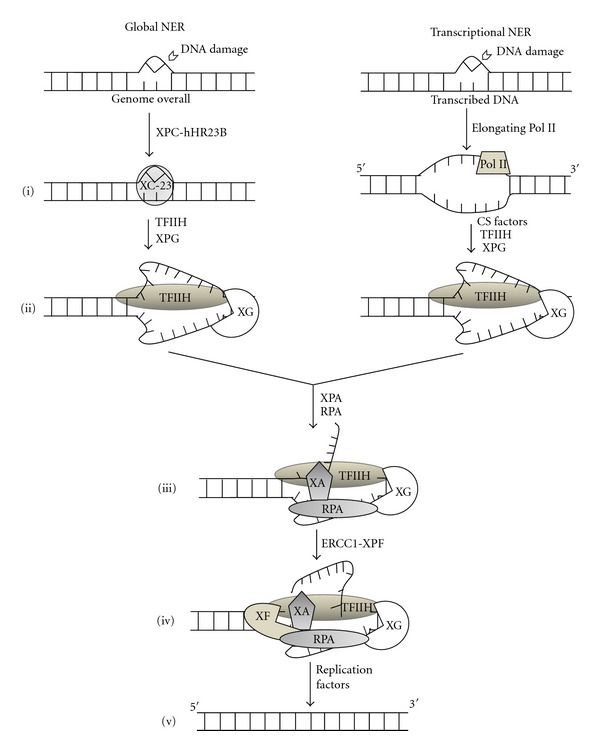
Molecular model of the NER system. Quoted from [7] and modified. (i) XPC-hHR23B (XC-23) binds and senses DNA distorting NER lesions in global NER, resulting in conformational alterations of the DNA. In transcriptional-coupled repair (NER), lesions are detected by elongating RNA polymerase II (Pol II). (ii) (left) XPC-hHR23B attracts TFIIH together with XPG (XG). TFIIH creates a 10- to 20-nucleotide open DNA complex. XPC-hHR23B is released. (right) CSA, CSB, TFIIH, XG, and possibly cofactors displace the stalled Pol II and then bind to the lesions. (iii) XPA (XA) and RPA bind and stabilize the open DNA complex. (iv) XG that is positioned by TFIIH and RPA cuts the damaged nucleotides at the 3′ site and ERCC-XPF (XF) that is positioned by RPA and XPA cuts them at the 5′ site. (v) DNA polymerase fills the gap and ligase seals the nick. Normal nucleotide sequence is consequently restored. Contacts drawn between molecules reflect reported protein-protein interactions.

DNA damage is recognized differently depending on whether the DNA is transcriptionally active (transcription-coupled repair) or not (global nucleotide excision repair: global NER) (Figure 2).

In global NER, a complex of xeroderma pigmentosum (XP) Type C (XPC) and human homolog Rad23B (hHR23B) detects the damaged lesion and recruits transcription factor II H (TFIIH, which is composed of a number of core subunits including XPB, p34, p44, p52, and p62 as well as cyclin-dependent-kinase-(Cdk-) activating kinase subunits including Cdk 7, cyclin H, and Mat1) to the lesion together with XPG. TFIIH that contains XPB and XPD helicases creates a 10- to 25-nucleotide open DNA complex around the lesion. XPA verifies the damage in this open DNA complex. Replication protein A (RPA) then stabilizes the open DNA complex and is involved in positioning XPG and excision repair crosscomplementing (ERCC1)-XPF endonucleases that are responsible for the DNA incisions. After removal of the damage-containing nucleotides, DNA polymerase fills in the gap and ligase seals the nick.

In transcription-coupled repair, Cockayne syndrome (CS) group A, CSB, TFIIH, XPG, and possibly other co-factors displace the stalled RNA polymerase II complex from the damaged lesion, which then becomes accessible for further repair process. After this initial recognition step, the damage is repaired in a similar manner to that observed for global NER.

Since the formation and persistence of DNA adducts of cisplatin leads to the development of apoptosis, an increased level of DNA repair consequently attenuates apoptosis progression. In order to remove the DNA adducts of cisplatin, to repair the DNA damage and to promote cell survival, the cell cycle is arrested. Once this occurs, then it is followed by NER. NER processing is thus associated with cisplatin resistance. In accordance with these data, increased expression of XPA, ERCC1, ERCC1-XPF complexes, and BRACA1 has been linked to cisplatin resistance [33–35].

### 4.4. Inhibition of Transmission of DNA Damage Recognition to the Apoptotic Pathway

The HER-2/neu protein plays an important role in mediating transmission of the recognition of cisplatin-induced DNA damage to apoptotic pathways. HER-2/neu is a transmembrane receptor with a tyrosine kinase domain in the cytoplasm, which has homology to the epidermal growth factor receptor (EGFR). HER-2/neu activation propagates down-stream signaling through the phosphatidylinositol 3-kinase/Akt (PI3K/Akt) pathway. G protein-coupled receptor (GPCR) agonists including various cytokines, angiotensin II and endothelin-1, induce EGFR and HER-2/neu transactivation [36, 37]. Cisplatin facilitates growth inhibition through activation of p21^Waf1/Cip1^ in a p53-dependent manner. The PI3K/Akt that is activated by HER-2/neu induces cytoplasmic localization of the CDK inhibitor p21^Waf1/Cip1^. A decrease in the nuclear level of p21^Waf1/Cip1^ abrogates the p53-dependent antiproliferative effects induced by cisplatin and consequently sustains cisplatin resistance [38].

## 5. A New Molecular Mechanism of Cisplatin Resistance

**Figure 3:**
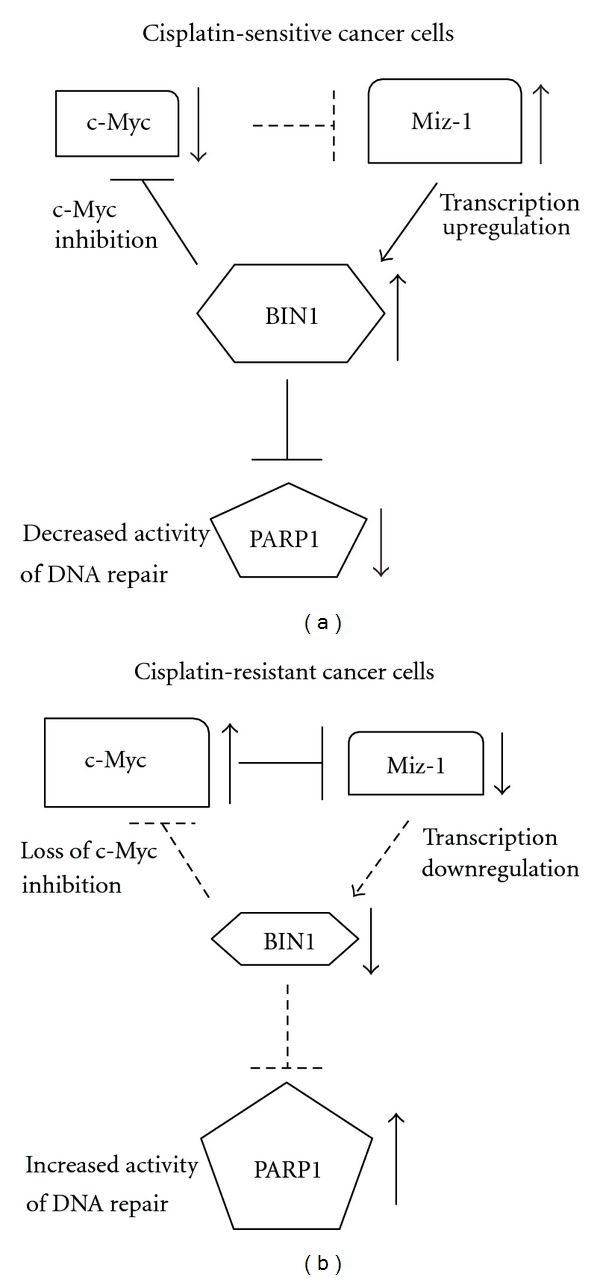
The molecular mechanism by which BIN1 is involved in cisplatin sensitization. Quoted from [5] and modified. The Miz-1-BIN1 interaction upregulates cellular cisplatin sensitivity by disruption of PARP1 activity. In cisplatin-sensitive cancer cells, a low level of c-Myc allows Miz-1 to stimulate BIN1 transcription, thereby maintaining a high cellular level of BIN1. The feedback inhibition of c-Myc by BIN1 perpetuates the decrease in c-Myc levels and results in decreased PARP1 activity, which consequently leads to downregulation of DNA repair activity. (b) In cisplatin-resistant cancer cells, c-Myc overexpression represses BIN1 expression by blocking the transcription activity of Miz-1. Loss of BIN1 feedback inhibition results in a robust increase in endogenous c-Myc and PARP1 activities, which consequently up-regulates DNA repair activity and cancer cell resistance to cisplatin. (a, b) Dashed lines indicate a decrease in the abundance or activity of a (positive or negative) regulator. Arrows indicate transcriptional up- or downregulation. Arrow size indicates the strength of this regulation.

A very recent investigation demonstrated a new mechanism by which the oncoprotein c-Myc enables cancer cells to acquire cisplatin resistance by suppressing BIN1, thereby releasing the DNA repair protein poly(ADP-ribose)polymerase (PARP) 1 [5] (Figure 3).

BIN1 was identified as a nucleocytoplasmic adaptor protein that can exert tumor suppressor properties by directly interacting with the c-Myc oncoprotein [39]. BIN1 is expressed in normal and benign cells and tissues but was undetectable in almost all estrogen receptor-positive or estrogen receptor-negative carcinoma cell lines. Complete or partial losses of BIN1 were documented in 60% of breast cancer tissue analyzed by immunohistochemistry or RT-PCR [40]. Reintroduction of BIN1 into human breast cancer and melanoma cell lines that lack its endogenous expression leads to loss of proliferation capacity and cellular death mediated by both p53- and caspase-independent pathways [40].

### 5.1. Involvement of BIN1 in Cisplatin-Sensitization

The molecular mechanism by which BIN1 is involved in cisplatin sensitization has become clear. Experiments involving forced depletion of the BIN1 protein using antisense or short hairpin RNA targeted toward *BIN1* in p53-positive, -null, -mutant cells resulted in increased cisplatin resistance. BIN1 interacts with c-Myc in the nucleus and inhibits its transactivation of target genes and cell transformation. Exposure of cisplatin-resistant cell lines expressing full-length BIN1 or a BIN1 deletion mutant lacking the MYC-binding domain (MBD) to cisplatin demonstrated that the MBD was essential for BIN1-mediated chromatin condensation and apoptotic cell death. However, addition of the c-Myc inhibitor 10058-F4, which disrupts the conformation of the c-Myc protein, to mimic BIN1-mediated inhibition of c-Myc was 25% less effective than full-length BIN1-expressing cells in inducing cisplatin sensitivity in an Annexin V-binding assay. These data suggest that BIN1 interacts with a non-Myc regulator protein to modulate cancer cell sensitivity to cisplatin.

### 5.2. BIN1 Directly Interacts with PARP1 at Its Automodification Domain and Inhibits PARP1 Activity by Blocking PARP1 Automodification Domain-Mediated Modulation of DNA Integrity

 A glutathione S-transferase (GST) pull down assay with the recombinant GST-tagged full-length BIN1 protein followed by tandem mass spectrometry of coprecipitating proteins identified PARP1 as a candidate BIN1 interacting protein. Immunoprecipitation of cell lysates with an anti-BIN1 antibody followed by Western blotting with an anti-PARP1 antibody demonstrated the association of BIN1 with PARP1. In addition, GST pull-down assays of DU145 prostate cancer cells using individual GST-fused domains of BIN1 and PARP1 showed BIN1 bound to the automodification domain of PARP1 through the BIN-amphiphysin-Rvs-related (BAR-C) domain of BIN1. The PARP1 automodification domain is known to increase in PARP1-mediated modulation of DNA integrity after DNA damage [41]. PARP1 is a key component of the base excision repair (BER) pathway and activated PARP1 recruits X-ray repair complementing defective repair in Chinese hamster cells 1(XRCC1), which acts as a scaffold for other BER-related proteins, DNA ligase III and DNA polymerase-*β*. Overexpression of BIN1 suppressed the poly(ADP-ribosyl)ation of histone H1 by 40%. Conversely, depletion of BIN1 increased histone H1 poly(ADP-ribosyl)ation. Immunoprecipitation of DU145 cells with an anti-PARP1 antibody followed by Western blotting with an anti-XRCC1 antibody demonstrated that BIN1 significantly abrogated PARP1-XRCC interaction. Moreover, single-cell DNA gel electrophoresis assays (comet assays) of DNA instability showed that overexpression of BIN1 increased DNA breaks and depletion of BIN1 inhibited DNA breaks, suggesting that BIN1 destabilized chromosomal DNA. Cells stably expressing the PARP1 N-terminal DNA binding domain (DBD), which disrupts the interaction between endogenous PARP1 and damaged DNA and acts as a PARP1-specific dominant negative inhibitor, attenuated PARP1 activity and the concomitant induction of cisplatin sensitivity, was sustained even in BIN1-defective cells. Thus, inhibition of PARP1 is indispensable for the induction of cisplatin sensitivity.

### 5.3. Overexpressed c-Myc Restores Intrinsic PARP1 Activity by Suppressing BIN1 Expression

c-Myc overexpression induces cisplatin resistance, whereas c-Myc inactivation increases cisplatin sensitivity. Using the c-Myc-estrogen receptor fused cell system, increased activity of c-Myc that was driven by 4-hydroxytamoxifen (4-OHT) robustly enhanced the poly(ADP-ribosyl)ation of histone H1, and cooverexpression of the PARP1 DBD abrogated this upregulation.

In addition, overexpression of c-Myc decreased BIN1 protein abundance and depletion of c-Myc increased BIN1 protein abundance. Thus, expressions of the *c-myc* and *BIN1* genes are inversely regulated. Furthermore, it is known that c-Myc represses the transcription of cell cycle arrest genes by a transcription initiator (Inr)-dependent repression mechanism [42]. A chromatin immunoprecipitation (ChIP) assay demonstrated that c-Myc binds to the BIN1 core promoter region through its Inr element. Miz-1, a Myc-interacting zinc-finger transcription factor, is known to bind to the Inr element of several genes that are repressed by c-Myc. Miz-1 functions as a counter partner of c-Myc and activates transcription of the genes that are repressed by c-Myc. c-Myc binds to Miz-1 at the Inr element and subsequently inhibits Miz-1-mediated transcription [42]. BIN1 promoter-driven luciferase activity was suppressed by cotransfection of Miz1 siRNA. In addition, depletion of Miz1 also decreased the expression levels of BIN1 mRNA and protein. A ChIP assay using an anti-Miz-1 antibody demonstrated that endogenous Miz-1 is recruited to the Inr-containing core promoter region of the *BIN1* gene in chromatin. Finally, depletion of Miz-1 decreased cisplatin sensitivity. These results suggested that Miz-1-induced BIN1 protein expression sustains the sensitivity of cancer cells to cisplatin.

## 6. Conclusion and Perspectives

**Figure 4:**
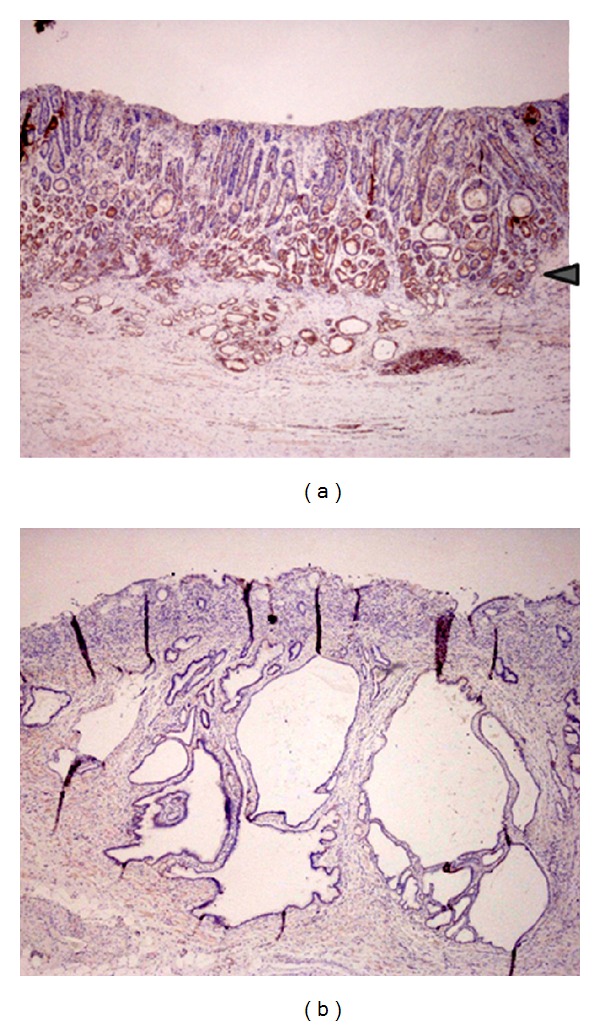
Immunohistochemical staining of BIN1 in a resected specimen of primary advanced gastric cancer. (a) BIN1-positive (arrowhead) and (b) BIN1-negative staining.

Extensive knowledge regarding the molecular mechanisms of cisplatin-induced apoptosis and particularly of cisplatin resistance is indispensable for the design of therapeutic strategies using cisplatin against intractable malignancies. It has been established that the mechanism of cisplatin resistance includes reduced drug uptake, increased drug inactivation, increased DNA adduct repair, and inhibition of the propagation of DNA damage signals to the apoptotic program. A novel mechanism mediating cisplatin sensitivity has recently been proposed, in which Miz-1-induced BIN1 protein expression sustains the sensitivity of cancer cells to cisplatin. We have found patients with advanced gastric cancer who are immunohistologically positive or negative for BIN1 expression (Figure 4). Thus, BIN1 may be a new potent marker for the prediction of cisplatin sensitivity, which can be used for the design of strategies for gastric cancer treatment. Furthermore, introduction of the BIN1 gene may also be a new therapeutic strategy for treatment of cisplatin-resistant gastric cancers. The rapid expansion in our knowledge regarding the molecular mechanisms of cisplatin resistance continues and will ensure that future anticancer treatment strategies can be devised and that the multifactorial mechanism of cisplatin resistance can be circumvented.

## Abbreviations

BIN1: Bridging integrator 1
MMR: Mismatch repair
HMG: Nonhistone chromosomal high-mobility group
TBP: TATA-binding protein
ATM: Ataxia telangiectasia mutated protein
ATR: ATM- and Rad3-related protein
MAPK: Mitogen-activated protein kinase
ERK: Extracellular signal-related kinases
JNK: c-Jun N-terminal kinases
MRP: Multidrug resistance-associated protein
ATF: Activating transcription factor
GSH: Gamma-glutamylcysteinylglycine
XP: Xeroderma pigmentosum
TFIIH: Transcription factor II H
Cdk: Cyclin-dependent kinase
RPA: Replication protein A
ERCC1: Excision repair crosscomplementing
CS: Cockayne syndrome
EGFR: Epidermal growth factor receptor
GPCR: G protein-coupled receptor
PI3K: Phosphatidylinositol 3-kinase
PARP: Poly(ADP-ribose)polymerase
MBD: MYC-binding domain
GST: Glutathione S-transferase
BAR-C: BIN-amphiphysin-Rvs-related
BER: Base excision repair
XRCC1: X-ray repair complementing defective repair in Chinese hamster cells 1
DBD: N-terminal DNA binding domain
4-OHT: 4-Hydroxytamoxifen
ChIP: Chromatin immunoprecipitation
Inr: Initiator.
